# Unidirectional Communications in Secure IoT Systems—A Survey

**DOI:** 10.3390/s24237528

**Published:** 2024-11-25

**Authors:** Lucian Gaina, Cristina Sorina Stangaciu, Daniela Stanescu, Bianca Gusita, Mihai Victor Micea

**Affiliations:** Department of Computer and Information Technology, Politehnica University of Timisoara, 300006 Timisoara, Romania; lucian.gaina@cs.upt.ro (L.G.); cristina.stangaciu@cs.upt.ro (C.S.S.); daniela.stanescu@cs.upt.ro (D.S.); bianca.gusita@cs.upt.ro (B.G.)

**Keywords:** security, Internet of Things (IoT), unidirectional communications, critical infrastructure, data diodes, network pumps, unidirectional gateways, unidirectional protocols

## Abstract

The security of Internet of Things (IoT) systems has consistently been a challenge, particularly in the context of critical infrastructure. One particular approach not yet employed in this domain is the unidirectional communication paradigm. This survey presents an analysis of the most prevalent unidirectional communication solutions, namely, data diodes, network pumps, unidirectional gateways, and unidirectional protocols. The objective of the survey is to present an analysis of the unidirectional communication methods that meet the requirements of IoT security. These methods are classified according to their implementation and operational mode. The survey analyzes the unidirectional communication solutions based on their performance, the level of security offered, the cost-effectiveness, and their cost of implementation. Additionally, it includes an analysis of the existing off-the-shelf unidirectional communication implementations found in the industry. Furthermore, it identifies some of the most important current issues and development directions.

## 1. Introduction

The widespread adoption of the IoT has transformed how people engage with technology, ushering in an era in which standard devices are seamlessly integrated to improve efficiency, convenience, and productivity. The core of this integrated network is communication, which involves data exchange between devices and systems.

While much of the research and development on the IoT has focused on bidirectional communication, there is a growing interest in using unidirectional communications, where data need to flow in just one direction. Unidirectional communication offers distinct advantages in specific IoT applications, mainly when simplicity, security, and efficiency are paramount.

Koohang et al. conclude, in [1], that as data security and privacy standards increase, so will the knowledge and awareness of IoT technology among users. Furthermore, implementing one-way communication methods will positively influence the increase in awareness and interest of users in using IoT systems.

### 1.1. IoT Security Issues

Some of the vulnerabilities identified in the IoT domain regarding data protection are weak security frameworks and solutions for connected devices or weak security measures and hardware parts owing to cost [2].

**Figure 1:**
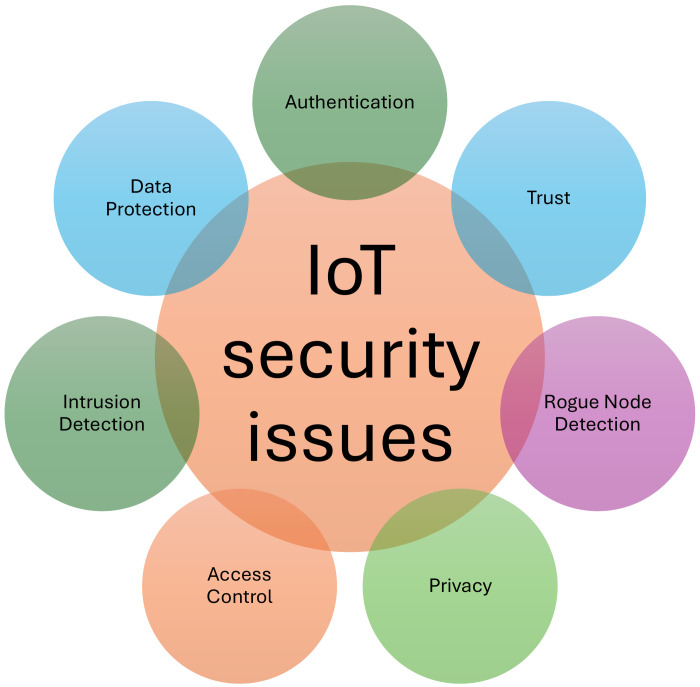
Summary of IoT security issues.

Figure 1 summarizes the current issues in the IoT domain. These issues have been identified and analyzed by Alrawais et al. in [3].

In the domains of healthcare [4] and Internet of Vehicles (IoV) [5], the data privacy and integrity issue remains unresolved and constitutes an open challenge. Given the vast quantity of data collected by IoT devices, securing these systems assumes significant importance if they are to be secure and feasible. The most prevalent attacks involve altering data during transmission, which endangers the safety of users (patients, drivers, passengers) by blocking safety and security systems. Integrating unidirectional communication methods into these systems can be employed to prevent the modification of data within them.

The sensitivity of medical data has arisen as a critical concern in an era involving technological breakthroughs and unprecedented access to information. The complexities of personal health information and the possible implications of its misuse highlight the crucial importance of preserving these data repositories. The dangers of tampering with or falsifying medical information are not only theoretical; they pose real concerns to individual well-being, healthcare systems, and the integrity of medical research [6].

According to [7], two security and privacy challenges from 6G-IoT have been identified. The growth of 6G-IoT poses new security and privacy risks, such as illegal data access, access network integrity concerns, and enterprise intelligence breaches. Due to third-party interference, satellite–UAV–IoT connections in space may suffer data privacy difficulties, affecting data exchange and transmission.

One key reason for using unidirectional communication in the IoT is the inherent security benefits. Unidirectional communication eliminates the necessity for bidirectional data flow, limiting the attack surface and the possibility of illicit access and data damage. This is especially important in critical infrastructure systems, where data integrity and confidentiality are valuable. In addition, healthcare systems and critical infrastructure were also listed in [8] as the sectors with the most significant potential to impact users in the event of an attack.

Furthermore, unidirectional communication improves scalability by minimizing network congestion to maintain bidirectional communication channels. This is especially true in large-scale IoT implementations involving dozens or millions of networked devices.

### 1.2. Unidirectionality of a Network

A network is referred to as unidirectional when all connections or information move in one direction only. This implies that within a unidirectional network, any link connecting two nodes can only transmit information in one direction. Consequently, no communication happens between them in the opposite direction.

To prove the concept of unidirectionality, let us consider a directed graph G=(V,E), where *V* is a set of nodes, denoted V={1,2,3,4,5,6,7,8,9}, and *E* is a set of edges, denoted E={(1,5),(1,6),(2,5),(2,6),(2,9),(3,4),(3,5),(5,6),(5,7),(7,2),(7,8),(8,3),(9,5)}. Each edge is a pair of vertices E⊆{(u,v)|u,v∈V}.

**Figure 2:**
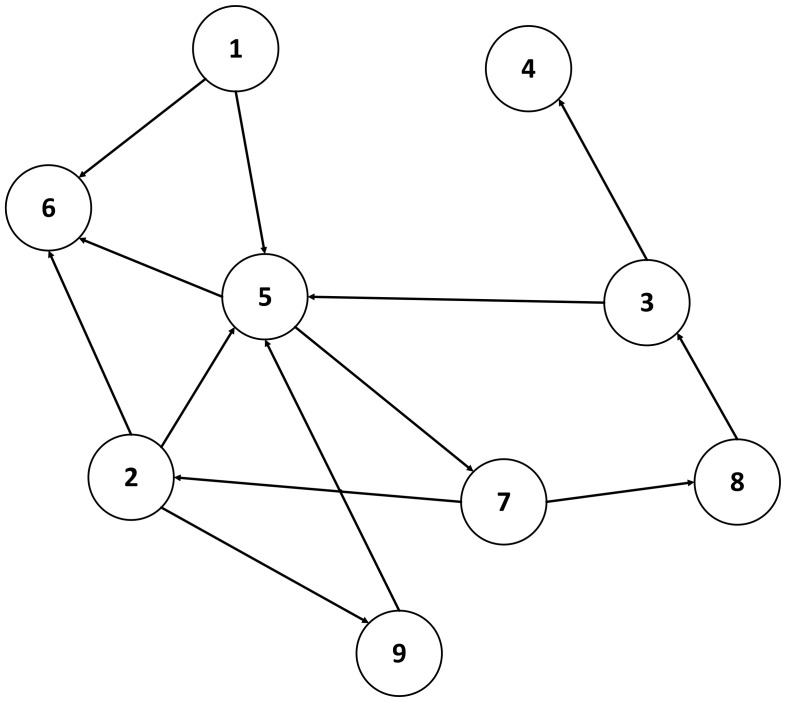
Unidirectional graph *G*.

Graph *G* from Figure 2 shows that the network described by this graph is unidirectional. For the purposes of this analysis, node one is considered the starting node. The information packet leaves node one and must reach node four without returning to the node from which it left. The Depth First Traversal (DFS) algorithm was used to traverse the graph.

The algorithm employs a stack in which the neighboring nodes are added. The current node and its neighboring nodes are traversed in stack order, and if the current node is the destination node, the algorithm terminates. If the stack becomes empty, it indicates no path between the source node and the destination node.

The traversal from node 1 to node 4 is conducted as follows:

- We commence by visiting the source node (1) and adding its neighbors (5 and 6) to the stack.
- Subsequently, node 6 is visited, and its neighbor node 5 is added to the stack.
- Subsequently, node 5 is visited, and its neighbors (2, 3, and 7) are added to the stack.
- Subsequently, the node at position seven is visited, and its neighbors at positions 8 and 9 are added to the stack.
- Upon visiting node 9, it was determined that it had no unexplored neighbors.
- Upon visiting node 8, it was determined that it had no unexplored neighbors.
- Subsequently, node 3 is visited, and then its neighbors, specifically nodes 4 and 8, are added to the stack.
- Finally, node 4 is visited and the target node is located.

Consequently, a unidirectional path is established between nodes 1 and 4, thereby indicating the existence of unidirectional communication within the network depicted in Figure 2.

### 1.3. Current Techniques

The most conventional methods of unidirectional communication are data diodes, network pumps, and unidirectional gateways. Data diodes are the most common unidirectional communication solution employed in critical infrastructure. A data diode is a physical network device or a unidirectional security gateway that allows outgoing data flow but prevents incoming data flow [9]. The hardware component of the data diode promotes physical unidirectionality, which only allows data to be transmitted from the source network to the destination network rather than the other way around [10]. The network pump was designed by Kang and Moskowitz, as documented in [11]. The goal of the network pump is to securely and reliably send messages from a low network to a high network. The pump underwent development to reduce the covert channel risk posed by the required message acknowledgments while maintaining system performance and reliability [12]. A unidirectional gateway is a network device or system that only enables data to be transmitted in one direction and prevents information from going back in the opposing direction. This technology guarantees the safeguarding of sensitive or classified data that are safely sent from one network to another, reducing the risk of illegal access or information leakage [13].

**Figure 3:**
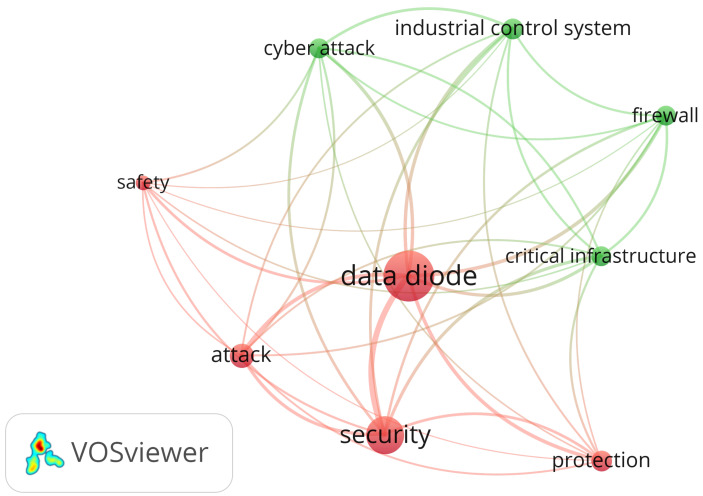
Data diode-related words and number of occurrences.

Data diodes are used in different fields of data protection. The graph in Figure 3, generated using the VOSviewer [14] tool, shows the areas where the data diode is used. The central node, designated as a “data diode”, and its associated connections suggest that it is a pivotal term within the given context, with the highest number of occurrences. The graph shows the most robust connection between nodes, which is between the data diode and security nodes. The strength of this connection indicates that the two phrases are frequently employed in the same context. The use of differently colored word clusters suggests the existence of subthemes or related areas within the same general theme. The dataset query was “data diode” and contained over 600 words. It was filtered to show words that occurred more than seven times in each research work.

**Figure 4:**
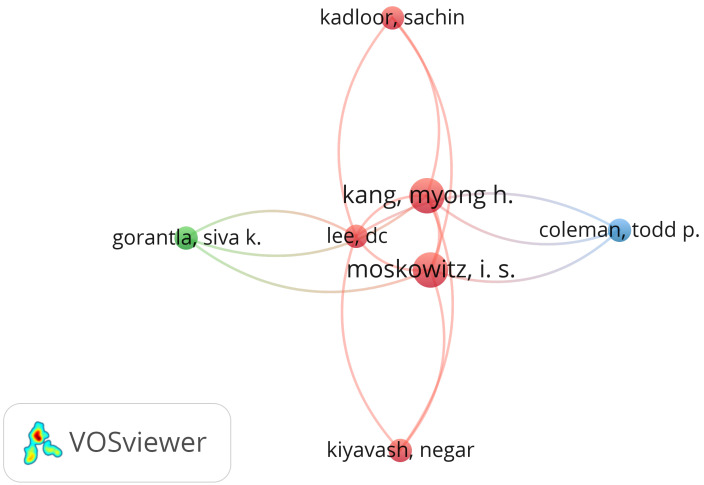
Network pump—most cited authors.

Figure 4 shows the most cited authors from the network pump field. These findings illustrate these individuals’ significance and impact within the field. According to the number of citations, the most cited authors are Kang, Moskowitz, and Lee. The red cluster (including Kang, Moskowitz, Lee, Kiyavash, and Kadloor) contains authors who are frequently cited together, which suggests a close relationship in the research field of network pump. The blue and green clusters (including Coleman and Gorantla) contain authors cited together across different areas or sub-topics of the main field. The query of the dataset was “network pump”. Out of 14 authors, only 7 met the threshold set as the minimum number of documents of an author, which was set as one, and the minimum number of citations of an author, also set as one.

**Figure 5:**
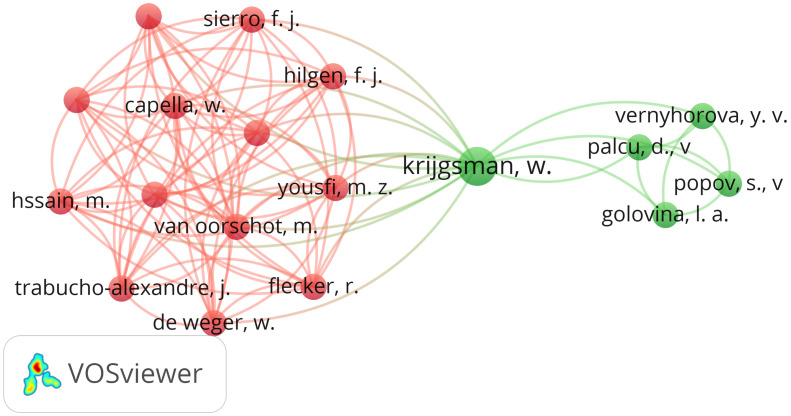
Unidirectional gateway—co-authorship teams.

Figure 5 shows the co-authorship network of the “unidirectional gateway” query. The analyzed data show two groups of researchers, the left group (red) and the right group (green), with Krijgsman in the middle as the only author who coauthored with both groups of researchers. This suggests that he is a pivotal figure, facilitating the integration of the two groups. The size of the Krijgsman node means that he has the highest number of occurrences. The researchers are grouped according to whether they have coauthored one or more papers.

**Figure 6:**
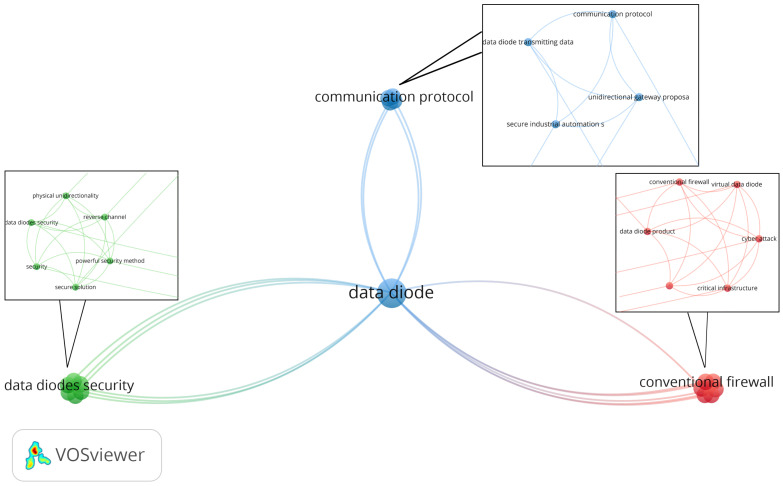
Unidirectional gateway—related domains and most used areas of development.

In Figure 6, the most researched domains and how these domains are used together are presented. It can be observed that the data diode is the new centerpiece, meaning the domain that is related to all other domains within the dataset. The dataset has been divided into three clusters based on the associations between domains (nodes). Cluster 1 (located on the right side and identified by the red color) consists of the following nodes: conventional firewall, critical infrastructure, cyberattack, data diode product, strict unidirectional gateway, and virtual data diode. Cluster 2 (positioned on the left side and identified by the green color) comprises the following nodes: data diodes security, physical unidirectionality, powerful security method, reverse channel, secure solution, and security. Cluster 3 (positioned at the top and identified by the blue color) comprises the following nodes: communication protocol, data diode transmitting data, secure industrial automation system, and unidirectional gateway proposal. The grouping within each cluster was based on the following criteria: the number of occurrences of the word, the number of associations, and the domains with which it is associated; thus, the nodes of clusters 1 and 2 have one occurrence and six associated nodes each, while the nodes of cluster 3 have one occurrence and four associated nodes. The diagram shows that the data diode is the central element among the three clusters, being associated with all other nodes and having a total of three occurrences.

Through this comprehensive analysis, we intend to show how unidirectional communication can improve the security, scalability, and efficiency of IoT deployments. Comprehending the benefits and downsides of unidirectional communication establishes the groundwork for building durable, long-lasting, and secure IoT systems that meet the evolving needs of the digital era.

### 1.4. Security Models

The Bell–LaPadula paradigm is a formal model [15] for imposing access control in government and military settings. It focuses on protecting data confidentially. The model is based on state machine theory and outlines the allowable actions in a given state. The Bell–LaPadula model defines two primary rules [16]:

1. **Simple security rule (no read-up)**: A subject cannot read information classified at a higher level than the subject’s allowed level of access.
2. Star (*)-property (no write-down): A subject cannot write data to a lower security level, thereby preventing the leakage of sensitive information.

Data diodes are designed to comply with the Bell–LaPadula security model’s principles, namely, the model’s *-property (also known as the “no write down” rule). However, their compliance is narrowly interpreted in the context of data flow compared to the usual read/write operations performed on objects by subjects. The following example illustrates this interpretation:

- Two networks (high-security network and low-security network) are considered, and a data diode connects them.
- One-way data flow (no write-down): The data diode is designed to permit the transfer of data from a lower-security-level network to a higher-security-level network. This design is directly compatible with the Bell–LaPadula model’s *-property, which restricts writing data to a lower security level to prevent data leakage.
- No read-up: The data diode physically prevents return data flow from the low-security network to the high-security network, avoiding the possibility of reading up. The simple security rule is intrinsically respected since data cannot move from one security level to another, ensuring that persons at a higher level cannot read data from a lower level.

Data diodes optimally implement the Bell–LaPadula model principles, particularly the *-property, by ensuring a one-way data flow from higher to lower security levels. This architectural design not only reduces the risk of unauthorized data disclosure but also precisely meets the model’s criteria. As a result, the data diode is an important and effective tool for ensuring data secrecy in high-security contexts.

### 1.5. Related Surveys

Recent research on IoT data and device security has revealed a growing gap in efficiently applying traditional security approaches to developing IoT applications. Security vulnerabilities in IoT applications have been categorized into two categories: difficulties with IoT device suppliers and the accessibility of resources and capabilities within IoT nodes. IoT sensor and device manufacturers increasingly focus on cost-cutting measures, often missing critical security. At the same time, the various natures of IoT applications, protocols, and hardware introduces a broader range of security concerns in IoT contexts [17].

To address these issues, newer security solutions for IoT devices with low resources have been created, employing robust machine learning (ML) techniques such as the TinyML framework. As explained by Dutta and Kant in [18], the core principle of integrating ML is to improve the agility of IoT nodes in safeguarding against evolving security risks.

By performing a comprehensive study on the convergence of 6G and IoT, Nguyen et al. in [7], investigate the rising potential presented by sixth-generation (6G) technology in IoT networks and applications. The most fundamental 6G technologies that will power future IoT networks are edge intelligence, reconfigurable intelligent surfaces, space–air–ground–underwater communications, terahertz communications, massive ultrareliable and low-latency communications, and blockchain. The authors of the study provide an in-depth discussion of the roles of 6G in a wide range of prospective IoT applications via five key domains, namely, healthcare IoTs, vehicular IoTs and autonomous driving, unmanned aerial vehicles, satellite IoTs, and industrial IoTs. Eavesdropping, hijacking, spoofing, and DoS attacks may occur in data communications and data management centers as the number of connections between devices and computing nodes at the network edges grows.

Lee’s study [2] extends existing privacy and vulnerability theories by emphasizing the importance of protecting physical privacy and user vulnerability in the context of home IoT setups. An empirical analysis containing 265 samples substantiated the suggested research paradigm. According to the research, user vulnerability significantly impacts privacy concerns and resistance to home IoT ecosystems. Furthermore, the study reveals differences in personal aspects related to vulnerabilities, privacy concerns, and reluctance to home IoT adoption. The principal vulnerabilities found were technology vulnerabilities, law vulnerabilities, provider vulnerabilities, and user vulnerabilities. Home IoT privacy risks have been discovered on smart TVs, smart speakers, smart plugs, IP cameras, smartphones, smart cars, and other devices that monitor personal life and activity habits or are remotely controlled.

Tariq et al., in [19], provide a comprehensive and clear review of the current state of IoT security anomalies and concepts, analyzing the main security issues related to IoT architecture, connectivity, communication, and management protocols. Additionally, a methodology for evaluating the effectiveness of solutions in different IoT use cases was developed. The security methods proposed in the study are those based on quantum security algorithms and those based on artificial intelligence (AI) and machine learning. The use of quantum-resistant cryptographic algorithms addresses the security issue of cryptographic algorithms that are not resilient against quantum algorithms; the integration of AI and ML algorithms enhances the capacity to safeguard data and mitigate the risk of business disruption in an attack. Furthermore, a method for transmitting data between IoT devices without access or interception, utilizing quantum key distribution (QKD), is also presented.

Tawalbeh et al., in [20], situate their work within the broader context of IoT security, elucidating the shortcomings of prevailing solutions, such as centralized architectures and layer-specific approaches, which prove inadequate in addressing inter-layer vulnerabilities. Previous studies have concentrated on the security of individual devices, the protection of personal data, or the implementation of isolated layer-specific solutions. However, these studies have often failed to consider comprehensive frameworks that facilitate seamless interoperability. The authors cite emerging solutions such as blockchain and AI-based anomaly detection as potential solutions to the scalability challenges inherent to resource-constrained environments. Building on these gaps, they propose a layered IoT security model that integrates cloud and edge components deployed in an Amazon Web Services (AWS) environment. This approach provides robust cross-layer security, seamless data transmission, and scalable privacy measures while addressing critical vulnerabilities of existing systems.

Kaur et al., in [21], cataloged and compared various attacks, datasets, and ML algorithm architectures for IoT device intrusion detection systems (IDSs). They also evaluated IoT datasets (containing security advantages and disadvantages) used for training models and identified key properties for their evaluation. Several issues were identified, including the diversity of communication standards and protocols, poor implicit device security, and the large volume of data generated by IoT devices. As a solution to these problems, the authors proposed using machine learning-based intrusion detection systems for attack detection and identification. Additionally, a list of unresolved issues was drawn up, the most significant of which was the use of IoT devices in different operating environments, making them more vulnerable to attacks they cannot withstand.

### 1.6. Contributions and Innovative Aspects

This study draws attention to the dearth of research on unidirectional communication methods in application domains increasingly prevalent in the current era. The IoT domain is one such area, and unidirectional communication methods have not been subjected to sufficient study and research within the context of the IoT. This represents a significant gap in the existing literature. In this context, this paper outlines the current research on unidirectional communications from the perspective of security in IoT systems and presents the following contributions:

- Highlights the lack of previous analysis on unidirectional communication methods, noting this as a narrowly defined field of study.
- Evaluates the state of research and development in unidirectional communications, focusing on solutions and their ability to meet IoT security needs and requirements.
- Provides a comprehensive literature evaluation, classifying unidirectional communication solutions based on security, reliability, and device type.
- Conducts a comprehensive analysis and categorization of the evaluation metrics employed by unidirectional communication solutions.
- Analyzes the areas of application where unidirectional communication methods have been used, presenting their utilization rate.
- Classifies commercial unidirectional communication products based on their application area, supported protocols, and covered attacks.
- Identifies a series of challenges and unresolved issues, summarizing potential research directions.

## 2. Unidirectional Communication Solutions and Their Classification

In today’s interconnected world, data and information flow security is critical, particularly in contexts where confidentiality, integrity, and reliability are essential. These technologies, which include data diodes, network pumps, unidirectional protocols, and unidirectional gateways, provide one-way data transmission pathways, thereby forming an impenetrable barrier to cyber threats and illegal access. This section delves further into these unidirectional communication technologies, their uses, and their essential function in protecting critical infrastructure and sensitive data. Through a comprehensive examination of their features and capabilities, this section aims to elucidate the significance of unidirectional communication solutions in modern cybersecurity strategies and their contributions to establishing resilient, secure, and future-proof network infrastructures.

**Figure 7:**
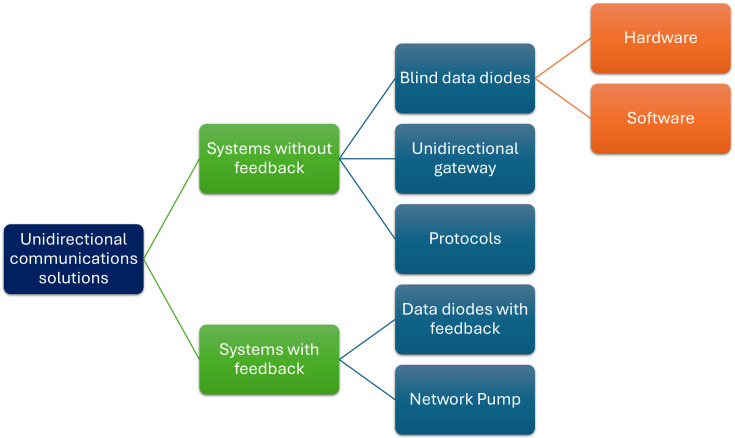
Classification of unidirectional communication solutions. Blind data diodes: hardware [6,24,25,26,27]. software: [28,29]. Unidirectional gateway [30,31,32,33]. Protocols [30,34]. Data diodes with feedback [35]. Network pump [12,36,37,38].

Unidirectional communication solutions have been categorized as shown in Figure 7, depending on their implementation.

### 2.1. Systems Without Feedback

#### 2.1.1. Blind Data Diodes

Data diodes are unidirectional network devices that allow data to pass only in one direction and block communication or data transfer in the other direction. This technology is essential for safeguarding sensitive information in critical infrastructure industries such as energy, finance, and government, where data integrity and confidentiality protection are of the utmost importance. By physically ensuring one-way data flow, data diodes reduce the possibility of cyberattacks, data breaches, and unauthorized access.

One important advantage is that hackers cannot remotely penetrate the data diode. Because data diodes rely on physical implementation rather than software for protection, they are designed to be highly secure. Additionally, they are frequently constructed with several security levels, such as access control and encryption [22].

Alternative methods of securing critical infrastructure, compared to the use of data diodes, present several disadvantages. Firewalls have a potential risk of attacks being launched by exploiting software vulnerabilities or misconfigurations. VPNs, even though they use secure communications, are susceptible to attacks because they use bidirectional communications. Air-gapping, while effective in isolating systems, introduces a major operational challenge by limiting real-time data access.

Operation technology (OT) devices are programmable systems for monitoring and controlling equipment, processes, and events, causing direct changes. They are most used in industrial systems, fire control systems, and physical access control mechanisms. Devices and services equipment, or interconnected systems to automatically acquire, store, analyze, and control data, are known as information technology (IT) devices. IT systems include computers, peripheral equipment controlled by a computer’s central processing unit, software, firmware, and related procedures, and services [23].

In this survey, data diode solutions are divided and analyzed from the perspective of their implementation; thus, the two categories are hardware-based data diode solutions and software-based data diode solutions.

#### Hardware-Based Solutions

This comparative analysis analyzes different methods for improving security using data diodes, a hardware-enforced unidirectional communication technology. The investigated solutions include hardware–software-integrated data diodes, encryption-augmented data diodes, AI-enhanced unidirectional communication, and data diodes for the security of photovoltaic (PV) systems.

A series of advantages and disadvantages of the hardware-based data diode solutions are synthesized in Table 1.

Khusanboyevich states, in [39], that the main disadvantage of hardware data diode devices is the low transfer rate. In cases where the data transfer rate is low, the data diode acts as a “bottleneck” for the secured system, which will not allow the information to be transferred in the proper time.

***Discussion***

In terms of performance, solutions such as AI enhancement and encryption integration provide advanced security but add complexity and possibly performance overhead. Hajal et al.’s solution from [6] is specifically developed to secure sensitive medical data transmission, ensuring compliance with healthcare standards while also protecting patient information. It includes an AI component for detecting non-compliant data, while Krause et al. proposed a solution in [26] suitable for defense in high-security industries that integrates data diodes, robust encryption, and a genuine random number generator, providing a robust and dependable solution for protecting sensitive data from illegal interception and data breaches. Hardware data diodes enable dependable unidirectional flow, but their low transfer rates make them difficult to use in high-data-rate scenarios, as Khusanboyevich states in [39].

The implementation complexity of advanced solutions that incorporate encryption or AI [6,26] means that they are more complex to develop and maintain than simple hardware–software approaches [24,25].

The integration of miniaturized data diodes in IoT devices proposed by Malatji et al. in [25] offers greater flexibility and scalability while providing robust security at the device level. However, this approach faces challenges due to inherent limitations in hardware data diode’s transfer rates, which can create bottlenecks in high-data-rate environments, affecting the timely transmission of information, as mentioned in [39].

The choice of an adequate data diode solution for Industrial IoT security is determined by the specific requirements, performance needs, and application scenarios. Hardware–software-integrated and miniature data diode solutions provide reliable and adaptable security, but encryption and AI-enhanced systems give advanced protection at the expense of higher complexity and potential performance difficulties. A thorough analysis of each solution’s strengths and weaknesses is required to ensure optimal security and performance in the intended application context.

#### Software-Based Solutions

Software data diodes are defined as a unidirectional communication solution for information transmission where the data transmission constraint is determined by the code and not due to hardware restrictions [39].

In the field of data diodes, most solutions have been implemented in hardware, resulting in a limited number of software solutions. Among the solutions analyzed, only two software-based data diodes that aligned with the objectives of this paper were found.

Table 2 summarizes the advantages and disadvantages of software-based data diode solutions.

According to Khusanboyevich, the main disadvantages of software data diodes are the higher complexity of the certification process of the solution and the theoretical possibility that data could leak out of a secure system through the reverse channel [39].

***Discussion***

Borges de Freitas et al.’s solution [28] takes a more versatile and adaptable approach to network security, making it appropriate for a wide range of applications and integration scenarios. On the other hand, the barcode-based solution proposed by Geiger in [29] is more specialized, with applications limited to scenarios requiring low data transfer rates. The software data diode using barcodes depends on the frames per second regarding data transfer limitations, so the PoC virtual data diode operates within the network infrastructure.

While the barcode-based solution may be more secure due to physical isolation, Borges de Freitas et al.’s distributed SDN-based approach improves resilience against system failures and network attacks by eliminating single points of failure. However, data diodes using barcodes potentially introduce vulnerabilities such as barcode duplication or interception.

Complexity and certification are issues for both methods. The distributed SDN-based solution necessitates extensive interaction with current applications and network topologies. In contrast, while more straightforward to install, the barcode-based approach encounters certification challenges due to its unconventional nature and the theoretical possibility of reverse-channel data leakage.

#### 2.1.2. Unidirectional Gateway

Unidirectional gateways, often called data diodes, represent a fundamental component in secure data transmission and network protection. In the context of cybersecurity, the concept of unidirectional data flow serves as a foundation for protecting critical information assets from cyber threats. Unidirectional gateways typically include hardware that establishes a strict one-way communication channel between two network domains, allowing data to flow solely in a single direction while preventing any return communication. Beyond the hardware, unidirectional gateways also incorporate software components that enable complex data management tasks, such as protocol conversion, data filtering, and real-time replication of data from the secure network to the external network. In this section, we delve into the principles, design considerations, deployment scenarios, and mitigations of existing unidirectional gateway solutions, elucidating their pivotal role in ensuring the integrity, confidentiality, and resilience of modern information infrastructures.

Table 3 compiles a range of pros and cons concerning the unidirectional gateway solutions.

***Discussion***

The security features of the solutions for improving cyber–physical system security have various strengths customized to different industrial contexts. The fiber-optic network communication solution proposed by Moussi Djeukoua et al. in [30] provides comprehensive protection via gateways and data diodes, assuring data integrity and forensic willingness through the use of solid communication protocols. Kim et al.’s UNIWAY solution from [31] focuses on secure file transfers with a modified FTP-based mechanism and fiber-optic communication, which ensures unidirectional data transfer via specialized transmitters and receivers. The OT-IT communication gateway from [32], proposed by Azarmipour et al., guarantees secure, unidirectional data transfer by utilizing an FIFO-based mechanism and a hypervisor for virtualization while keeping OT and IT systems separate. Finally, in the unidirectional gateway method in [33], Lin et al. combined hardware and software to establish a secure, unidirectional data transmission line, while virtual routing and forwarding technology offers a versatile and cost-effective solution for smaller systems.

When evaluating the implementation complexity of solutions, the fiber-optic network communication method has the highest complexity since message repetition and encryption are required to compensate for the lack of acknowledgment in data diodes. Both the UNIWAY system and the OT-IT communication gateway present moderate complexity. The UNIWAY system involves setting up proxy servers and specific fiber-optic connections for secure file transfer. In contrast, the OT-IT gateway uses FIFO techniques and hypervisor technology to ensure unidirectional data flow. The changeable complexity of unidirectional gateways makes them a flexible option with the potential for decreased complexity through the use of VRF technology, which is especially useful for smaller systems with limited resources.

From a financial standpoint, the most cost-effective solution for large-scale systems is an OT-IT communication gateway. This can be accomplished using FIFO methods and virtualization via a hypervisor, even if it requires a significant investment in virtualization technology. On the other hand, unidirectional gateways provide a flexible pricing structure, being a more inexpensive and technically more accessible alternative, which is especially useful for systems where standard unidirectional gateways are prohibitively expensive. Because of the extensive technology and encryption required, the fiber-optic network communication technique is the most costly on the list. It is also noted for its powerful security features, which use gateways and data diodes. The relatively moderate prices of UNIWAY are determined by the length of the fiber-optic infrastructure required for its file transfer procedures.

Each solution has distinct strengths and challenges specific to particular industrial objectives and security requirements. Security needs, implementation complexity, and financial limits should all be considered when determining the best solution. Fiber-optic network connections and UNIWAY provide strong security for scenarios with significant risks, whereas the OT-IT gateway and VRF technology provide adaptable and cost-effective solutions for a wide range of operations.

#### 2.1.3. Unidirectional Protocols

Unidirectional protocols are an essential paradigm in IoT security. They allow safe one-way data transport while preventing information backflow. Designed as software solutions, unidirectional protocols play an important role in protecting sensitive networks from cyber threats such as virus exposure, malicious attacks, and data exfiltration efforts by building a solid mechanism for information flow.

The low number of unidirectional protocols is due to technical difficulties, high communication costs, and the need for significant adaptations of existing protocols to work efficiently in such networks. Of the solutions studied, only two unidirectional protocols meet the analysis objective in this paper, as presented below.

The benefits and drawbacks of the protocols are summarized in Table 4.

***Discussion***

The solutions listed above discuss various communication protocols and target different aspects. Sharma et al.’s review [34] evaluates IoT data protocols such as MQTT and CoAP, whereas Moussi Djeukoua et al.’s solution review [30] delves into protocols like LLC1 and LLC2 for secure communication in CPS.

Sharma et al.’s protocol comparison emphasizes MQTT and CoAP’s light weights and outstanding performance, making them suitable for low-latency applications. Moussi Djeukoua et al. focus on the dependability and security of communication protocols, ensuring data integrity and forensic preparedness in CPS systems.

In summary, while both solutions address communication challenges in the IoT and CPS, they cater to different applications and priorities. Sharma et al. focus on efficiency and latency in smart home automation, while Moussi Djeukoua et al. prioritize security and reliability in CPS environments.

### 2.2. Systems with Feedback

#### 2.2.1. Data Diode with Feedback

Genua GmbH’s cyber-diode [35] incorporates various security features into a robust industrial-grade device. Its highly secure design is built on a minimalist and hardened OpenBSD operating system, which uses the L4 microkernel to generate separated data extraction and data provisioning environments. The cyber-diode’s architecture ensures minimum attack surfaces and strong security against cyberattacks.

One of the most significant breakthroughs in cyber-diode technology is introducing a feedback mechanism that ensures data delivery. In contrast to standard data diodes, the cyber-diode has a limited feedback channel that sends a status bit to validate data reception. This technique enables error-free transmission and maximum data throughput, with speeds up to 1 Gbit/s [35].

The cyber-diode may support a variety of protocols, including OPC UA, FTP, SMTP, TCP, UDP, and Syslog. Its adaptability allows it to be used in various industrial applications. The cyber-diode supports the Industry 4.0 standard OPC UA, which facilitates secure and reliable communication between field-level sensors and cloud applications [35].

The data diode, with feedback provided by Genua, marks a significant leap in industrial data diode technology. It offers an effective solution for the secure monitoring and optimization of essential plants and processes by combining strong security measures, a dependable feedback mechanism, and compatibility with modern protocols.

#### 2.2.2. Network Pump

The Naval Research Laboratory (NRL) created the network pump to provide a secure and dependable communication route between applications with different levels of security. Over the years, numerous implementations and theoretical studies have been carried out to improve its performance and security.

In the context of securing critical infrastructure, a network pump presents several advantages over alternative security methods. Compared to a firewall, a network pump is a hardware-based solution with predefined functionality. This eliminates the risk of misconfiguration and reduces vulnerabilities. In contrast to VPNs, a network pump enables the implementation of a controlled one-way data transfer, thereby preventing external access and data exfiltration, even in the event of an intermediary device being compromised. In contrast to intrusion detection/prevention systems, which merely detect threats, this system’s unidirectional architecture completely prevents external access. Compared to data encryption solutions, it offers protection without additional encryption by means of physical isolation of networks and the prevention of unauthorized access at the hardware level.

Table 5 presents various advantages and disadvantages related to network pump solutions.

***Discussion***

Referring to the reliability of the systems, in [12] Kang et al. proposed a method that prioritizes hardware reliability and includes battery backup to prevent illegal data transitions between high- and low-security levels. In [37], Gorantla et al. focus on theoretical assurance against covert channels, especially the issue of potentially encoding information in acknowledgment timings, to improve high-user reliability. The prototypes used in [36] demonstrate the practical reliability of their ideas across multiple layers and platforms. The process acknowledgments on the E-Pump were used at the process layer, while the TCP acknowledgments on the transport layer were employed on the D-Pump.

Gorantla et al., in [38], present the most comprehensive analysis of covert channels, focusing on message control time and inserted noise. Kang et al., in [12,36], also address this issue by incorporating practical implementations and noise injection into acknowledgment durations to mitigate covert channels.

Kang et al. prioritize fairness and DoS prevention to ensure that the pump can manage multiple senders and recipients without favoritism while protecting against attacks. These aspects are also considered in the prototype designs of E-Pump and D-Pump.

Each approach to the network pump provides distinct perspectives and solutions to the issues of safe and fair communication at varying security levels. Kang et al. offer a detailed review and practical improvements, while Montrose and Parsonese’s prototype implementations provide real-life examples of the pump’s adaptability. Gorantla et al.’s information-theoretic approach provides strict security against covert channels. Collectively, these studies provide a thorough overview of the network pump’s capabilities and prospects, emphasizing its importance in secure communication systems.

## 3. Performance Evaluation and Metrics

**Figure 8:**
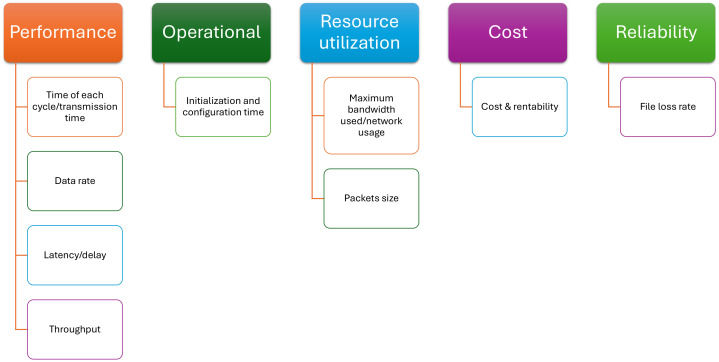
Analysis of metrics used in evaluated solutions. Performance: Time of each cycle/transmission time [24,34], Data rate [3,24], Latency/delay [6,24,28], Throughput [6,27]. Operational: Initialization and configuration time [24,34], Resource utilization: Maximum bandwidth used/network usage [6,28], Packets size [3]. Cost: Cost & rentability [6,25,27]. Reliability: File loss rate [28,40].

A synthesis of the most popular metrics used for performance measurement of unidirectional communication solutions analyzed in this work was made. The metrics are explained below and depicted in Figure 8, grouped by their usage.

**Initialization and configuration time** represents the period of time for the initial setup and configuration of a system (e.g., diode) before it can start operating.
**Time of each cycle/transmission time** is the time period required for a cycle of operations to be completed or for a packet to be sent from source to destination.
**Data rate** is represented by the number of bits that can be processed and transmitted per time unit [3] (usually, the second is used as a time unit).
**Latency/delay** is the time period when the package is sent and when it reaches its destination [6].
**Maximum bandwidth used/network usage** represents the maximum amount of data that can be used for transmission during a time interval.
**Cost and rentability** are defined as the cost of implementation, operation, and maintenance of a solution compared to its benefits and cost effectiveness relative to other existing solutions.
**Throughput** means the amount of data that the system successfully processes and transfers in a given time.
**Packet size** is the quantity of data in the transmitted packet, measured in bits [2].
**File loss rate** (loss file/send file) is the ratio of the number of lost files to the total number of sent files [40].

Following a detailed analysis of the metrics identified in the literature on unidirectional communication solutions, it was found that this area has not been sufficiently explored. The metrics discussed highlight the performance, operability, efficiency, and costs associated with unidirectional communication methods. Each metric is designed to evaluate a specific aspect of the system, and their interpretation can assist in determining the suitability of a security method for a particular purpose. This set of analyzed metrics is relevant and useful in evaluating a unidirectional security method. Our analysis, synthesized in Figure 8, demonstrates that each unidirectional communication method described in the articles uses evaluation metrics specific to that implementation. This reveals that there is currently no universal set of unidirectional communication evaluation metrics that apply to all types of one-way communication. This highlights the need to develop a uniform evaluation framework that ensures rigorous and consistent comparability between the different solutions proposed in this area.

## 4. Areas of Application

**Figure 9:**
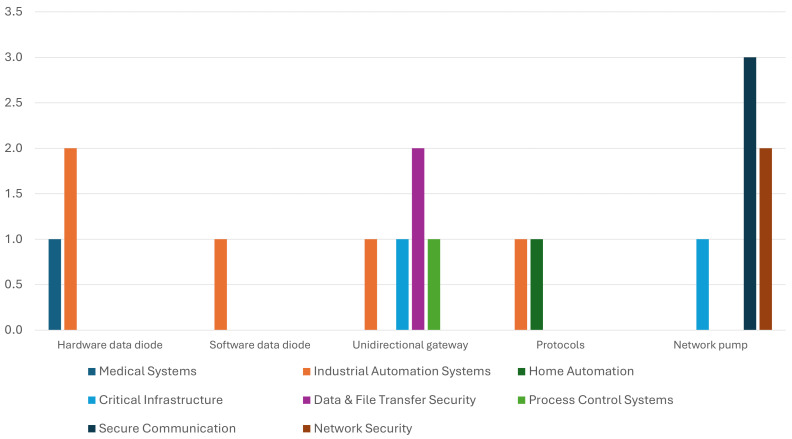
The use of unidirectional communication solutions in application areas.

This section analyzes the application areas of the unidirectional communication methods studied and discussed in Section 2. The classification presented in Table 6 highlights the areas in which unidirectional communication methods have been used and provides insight into their potential for use in areas where they have not yet been employed. Figure 9 illustrates the extent to which the various unidirectional solutions were employed in the identified application areas, as outlined in Table 6.

As evidenced by Table 6 and Figure 9, the most prevalent areas of application of unidirectional communication methods thus far have been in industrial automation systems and critical infrastructure. This suggests that there are potential avenues for extending the implementation of unidirectional communication methods to other application areas, as outlined in Table 6.

## 5. Unidirectional Communication Products

This section analyzes the most commonly used commercial data diodes and describes the technical aspects of each evaluated solution. Table 7 displays a proper analysis of commercial data diodes, highlighting which protocols are supported, which threats the diode can defend against, and which domains these solutions are employed in.

### 5.1. Waterfall Security

#### 5.1.1. WF-600

This data diode is immune to network threats, delivering unrivaled security for vital systems. Unlike traditional firewalls, it ensures a more robust defense by allowing safe IT/OT integration at the criticality boundary. The all-in-one platform combines proprietary hardware with the Waterfall OS to provide a highly optimized environment for native connection functioning. The solution offers extensive performance with throughput ranging from 1 to 10 Gbps and flexible configurations to satisfy any network requirement. The system ensures proven dependability with a standard high-availability (HA) option that eliminates single points of failure. Furthermore, the data diode offers cloud connectivity, allowing users to take advantage of the most recent automation developments without exposing their operations to ransomware threats.

#### 5.1.2. WF-500/WF-500 DIN Rail

This solution has a modular design that allows for simple customization and upkeep. It has a typical throughput of 1 Gbps and the capability of multi-Gbps rates via multiple TX/RX pairs. The connections on the front panel enable obvious system visibility, and it supports a wide range of commercially available off-the-shelf (COTS) software connectors, removing the need for costly modification fees. Furthermore, the flexible connection hosting on the hardware is compatible with all major operating systems, ensuring adaptability and compatibility for various use cases.

#### 5.1.3. WF for Intrusion Detection Systems (IDSs)

The system provides secure port mirroring from OT networks to IT networks, removing the possibility of introducing internet-based cyber risks to monitored networks. To simplify the management of IDS sensors, the network sensors may be effortlessly put on IT networks for easy management, all while maintaining the safety of monitored OT networks. This solution integrates seamlessly with the security operations center (SOC) while the system enables both stand-alone operations and integration with industry-leading SIEM/SOC systems. Furthermore, it is intended to be adaptable, with hardware configurations that avoid the need to introduce new hosts or software into critical industrial control system (ICS) networks.

### 5.2. Owl Cyber Defense

#### 5.2.1. Owl Perimeter Defense Solution-1000 (OPDS-1000)

This solution provides performance and security with three configurations: basic capacity, at 26 Mbps; mid-capacity, reaching 155 Mbps; and high capacity, offering 1000 Mbps. The system has a wide range of applications that serve various needs, such as processing sensor data and real-time database historical information and protecting vital infrastructure from ever-increasing external threats.

#### 5.2.2. XD Verge

Thanks to FPGAs and hardware isolators, the XD Verge solution excels at packet filtering and one-way data transport. It ensures no routable information is transferred between the source and destination networks. With 1 Gbps speed and very low latency, this solution outperforms CPU-based competitors by up to 150 times. The receiver FPGA reconstructs the contents of the packets, maintaining data integrity. Furthermore, the system incorporates packet-by-packet whitelist content filtering, with non-compliant packets being rejected by the source-side FPGA before passing through the hardware isolator.

#### 5.2.3. XD Prism MPP

This technology offers robust support for multi-protocol one-way data flows, including UDP- and TCP-based transfers, with a maximum throughput of up to 10 Gbps. It has a 1U form factor and minimizes size, weight, power, and cost (SWaP-C). It offers top-tier security using NSA/NCDSMO-approved data diode components (Owl V7 Communication Cards). Furthermore, its STIG-compliant, CLIP-enforced operating system strengthens its security credentials. This hardware-based solution is significant for high-security-data transmission applications because it enables Raise-the-Bar (RTB)-compliant cross-domain solutions.

### 5.3. OPSWAT—NetWall USG

NetWall USG has features that provide reliable data delivery and data loss protection. It ensures data loss-free payload delivery thanks to anti-overrun control, which successfully prevents data overflow, retransmissions, and synchronization difficulties. The system uses a one-way data flow enforced by a secure, non-networked serial connection to ensure data integrity. The solution is easy to deploy because it comes preconfigured for quick and seamless setup, excluding the need for complex firewall audits or configurations. This ease of use extends to its scaling possibilities, with 1 Gbit or 10 Gbit throughput options that may be software-selected based on user requirements. Regarding transfer rates, it offers options from 50 Mbit/sec to 1 Gbit/sec, 10 Gbit/sec, and even 10 Mbps to 50 Mbps (Din Rail). This solution provided by OPSWAT protects critical infrastructure and industrial environments against industrial assault methodologies detailed in MITRE ATT&CK for ICS.

### 5.4. FOX-IT—Fox Data Diode

Fox data diode is a high-performance platform that provides organizations with lightning-fast data transfers, impeccable security, and industry-leading certifications. The transfer speeds range from 1 to 10 Gbit/sec, quickly transmitting massive datasets in milliseconds. The platform has acquired prominent certifications such as CC EAL7+ and NATO’s TOP SECRET and Green schemes, independently recognized by industry experts.

### 5.5. Siemens—Siemens DCU

This is a vendor-neutral and machine-independent system that interfaces easily with all industrial systems. This technology ensures effective and dependable data acquisition with a data transfer speed of up to 200 Mbit/sec. One of its distinguishing aspects is its simple maintenance, which allows for uninterrupted functioning. The system offers a local data buffer through a USB drive to improve data accessibility and security, ensuring that sensitive data is always accessible. Furthermore, the data are collected using standard protocols provided by OWG software.

### 5.6. Fend Incorporated

Fend’s advanced diodes are innovative solutions with powerful features designed to protect important data and network infrastructure. The most notable features are DoS attack protection, anti-tamper protection, power loss protection, configurability provided by Fend’s Diode Configuration Tool, secure data hosting, complete optical isolation, and encrypted protocol options for data in transit. The maximum data throughput of Fend’s advanced diodes ranges from 5.0 Mbps to 15.0 Mbps.

### 5.7. Arbit

This data transfer solution comes in two configurations, with 1 GbE and 10 GbE data transfer speeds. It has NATO COSMIC TOP SECRET and EU TOP SECRET communication accreditation, making it suited for the most sensitive and confidential data transmissions. With no maximum file size restriction, the capacity of this system is only limited by the available disk space on proxy servers. It has 64 data channels per diode, suitable for efficient data transport. One of its distinguishing features is the ability to prioritize data channels on a transactional basis, ensuring that vital information is prioritized. It also supports up to 24 streaming channels for various sorts of data, such as logs, video, and radio through UDP. It provides high availability with peer-to-peer recovery, ensuring data access even in the case of unforeseen disruptions.

One potential application of unidirectional communication is in the context of an urban traffic monitoring system, particularly in areas of high traffic density or critical intersections. Monitoring systems comprise various devices, including video cameras, traffic sensors, weather measuring instruments, and other equipment that collect data of significant importance to understanding road traffic patterns. The data are transmitted to a central system, where they are analyzed and processed. Therefore, unidirectional communication plays an essential role in ensuring the security and integrity of the system during data transmission. In a bidirectional communication scenario, an attacker can gain control of devices, manipulate traffic data, or even compromise the functionality of critical elements such as traffic lights. Such a breach could significantly disrupt the flow of traffic, including the potential for severe congestion and accidents. The rationale behind the necessity of unidirectional communication in this system is threefold: firstly, to prevent the manipulation of peripheral devices, such as traffic lights, control cameras, and sensors; secondly, to safeguard the integrity of the data flow; and thirdly, to guarantee the safety of road users, whether drivers or pedestrians. An instance of a device capable of implementing unidirectional communication in this traffic monitoring system is the Siemens DCU data diode, which is utilized in the transportation industry, as evidenced in Table 7.

**Table 7:** Analysis of data diode commercial products.

Producer	Model Name	Supported Protocols	Covered Attacks	Areas of Application	Ref.
**Waterfall** **Security**	WF-600	NS	Remote attacks, malware, DOS attacks, ransomware, human errors from breaching the protected network	Electrical power plants, gas and oil industry, rails, water utilities, manufacturing, facilities, mining metal, hydropower-generating utilities	[41,42]
	WF-500/ WF-500 DIN Rail	FTP, SMTP, SNMP Traps, Syslog, RSV, OSIsoft PI, Modbus, WMQ, eDNA, ICCP, OPCDA	Targeted attacks, secure enterprise-wide visibility, safe remote access		[43,44,45]
	WF for IDS (INTRUSION DETECTION SYSTEMS)	NS	Remote attacks, malware, DOS attacks, ransomware, human errors originating on external networks from compromising or impairing industrial operations		[46,47]
**Owl** **Cyber** **Defense**	Owl Perimeter Defense Solution-1000 (OPDS-1000)	TCP, UDP, Syslog, RFTS, SNTS, SNMP Traps, SMTP, FTP	NS	NS	[48,49]
	XD Verge	UDP, ARP (Source Side)	NS	NS	[50,51]
	XD Prism MPP	UDP, TCP-based file transfers or data streams, Utilize Owl RFTS	NS	NS	[52,53]
**OPSWAT**	NetWall USG	FTP, SFTP, folder and file transfers/copying, SMB, CIFS	MITRE ATTCK for ICS	NS	[54,55]
**Fox-IT**	Fox Data Diode	TCP, UDP, SMB, FTP, SCP	NS	NS	[56,57]
**Siemens**	Siemens DCU	NS	NS	Transportation industry	[58]
**Fend** **Incorporated**	SE15	FTP, FTPS, TCP, UDP, Modbus TCP, Modbus RTU, BACnet (in), LON-IP (in)	DOS attacks	Manufacturers, oil and gas, water treatment, electric infrastructure	[59,60]
	SE5				[61,62]
	XE5				[62,63]
	CE5	FTP, FTPS, TCP, UDP, Modbus TCP, BACnet (in), LON-IP (in)			[62,64]
	XE15				[60,65]
	CE15				[60,66]
**Arbit**	The Arbit Data Diode 10 GbE	SMTP, FTP, SFTP, SMB, NFS, NTP, Streaming (TCP, UDP), REST API Forwarder (HTTP, HTTPS)	NS	NS	[67]

**NS:** not specified.

## 6. Open Issues

The IoT is a concept that proposes a space where smart devices communicate and collaborate to improve efficiency, convenience, and quality of life. However, as the IoT expands, new technical challenges and unresolved issues arise, especially in the area of unidirectional communications. This section, therefore, presents the main issues and challenges discovered during our research. It provides a summary of the open issues identified by the authors with regard to the unidirectional communication solutions presented in Section 2.

Improvements are needed for data diodes, which are essential for maintaining the physical unidirectionality of data flow to facilitate the transfer of various types of data over Ethernet channels [24]. There are difficulties in the full physical realization of data diodes on FPGA boards, which calls for more network engineering research [24,25]. To reduce the risk of data loss, it is essential to address unidirectionality’s incapacity of acknowledging data packages [68]. Additionally, the rise in edge computing has impacted IoT adoption, sparking greater interest across a range of industries [17]. As 6G-IoT develops, new security and privacy issues like illegal data access and edge intelligence breaches arise [69]. Deploying satellite–UAV–IoT connections in untrusted situations also gives rise to issues about privacy leakage during data transmission and exchange [7].

Challenges continue to arise within network pumps when the optimal buffer size and transmission rate are determined for optimal and efficient data transmission. Research in the direction of a “quantum pump” seeks a definitive analysis, but no clear-cut solution has surfaced despite efforts to examine the statistical covert channel in the pump. The absence of an exact solution is regarded as a mathematical quirk rather than an essential consideration for proper design decisions. The network pump shows a covert channel issue from connect/disconnect messages, particularly in cases when the user-specified connection parameter has no per-unit time limitations [12].

Due to resource limitations, designing secure and effective protocols is still a significant challenge in IoT. Due to protocols relying on wireless packet transmissions, many current protocols use significant resources. It is essential to balance performance, energy consumption, and security requirements [3,70]. Furthermore, it is crucial to assess suggested solutions for resolving vulnerabilities like those impacting CoAP [70]. To develop acceptable and effective security solutions for IoT devices, collaborative security approaches, including all stakeholders, are required [71].

Unidirectional gateways face challenges in minimizing the risks of cyberattacks and reaching confident security criteria for commercial solutions [30]. There is plenty of opportunity for additional research and improvement when it comes to applying unidirectional data transfer to other protocols like MODBUS, DNP3, and OPC [72]. Additional efforts should focus on defining the specifics of the flow of information for purposes of feedback and validation [32].

The analysis conducted in this paper has enabled the identification of some areas of interest and challenges that have not been previously mentioned or summarized in the literature. These findings offer a novel perspective on the field of study, highlighting existing gaps and suggesting potential directions for future research.

1. Data security and confidentiality
  - In unidirectional communications, devices transmit data without receiving acknowledgment from the destination, making it difficult for attackers to detect data interception or alteration. Solutions to this problem include strong data encryption, but implementing these solutions on resource-constrained devices remains a challenge.
2. Transmission reliability
  - Unidirectional communications do not allow acknowledgment of data reception, so lost packets cannot be retransmitted. Faulty error coding methods (FEC) and data redundancy can be used to alleviate this problem, but they may increase power consumption and bandwidth requirements.
3. Power management
  - Energy-efficient communication is essential for IoT devices, especially battery-powered ones. Unidirectional communication should minimize energy use for transmission and use communication protocols that allow devices to save energy.
4. Scalability
  - Collecting and processing data from several unidirectional IoT devices necessitates algorithms capable of handling huge amounts of data and providing usable results in real time. These solutions must be resource-efficient while maintaining the integrity and security of the aggregated data.
5. Interoperability
  - The absence of a unified set of metrics prevents a comprehensive validation of unidirectional communication security methods for IoT devices and systems.
  - The lack of common standards among different IoT device manufacturers can lead to interoperability and integration issues between various systems. Adopting open standards is essential to ensure interoperability.
  - Choosing and implementing appropriate communication protocols for different unidirectional IoT applications can be complicated. Protocols must be efficient in terms of power consumption and latency and compatible with other networks and devices.
6. Network configuration and management
  - Identifying and fixing problems in IoT devices that use unidirectional communication is difficult without a second communication channel. Solutions may include regularly monitoring device status and using anomaly detection algorithms to identify problems without requiring direct feedback.

Overall, improving the security, effectiveness, and dependability of unidirectional communication in IoT contexts depends on resolving the open concerns in data diodes, network pumps, protocols, and unidirectional gateways.

## 7. Conclusions

This work presents a structured study on the advances in unidirectional communications, emphasizing their significance and usefulness in ensuring the security, scalability, and efficiency of IoT systems. To date, there has not been published a detailed analysis of unidirectional communication methods and their ability to meet the security requirements of IoT. As it stands, this is still a relatively narrow field of study, but with a high potential for impact in the following years.

Our survey reviews and categorizes the recent relevant literature on the unidirectional communication solutions, including data diodes, network pumps, unidirectional protocols, and unidirectional gateways. These solutions are analyzed and classified based on perspectives such as security, reliability, and device type. The advantages and limitations of each approach are outlined and discussed, to help improve understanding of the complexity and diversity of these methods.

A comprehensive analysis of the evaluation metrics employed by unidirectional communication solutions has been conducted. As a result, the existing metrics are categorized based on their usage. Further, we have investigated the areas of application where these methods have been used, presenting their utilization rate.

We have also evaluated and categorized the existing off-the-shelf products that have unidirectional communication capabilities. Their application spectrum, protocol support and resilience against different cyber threats has been analyzed. This work compiles our findings to provide a foundational summary of unidirectional communication technologies, offering insights that could propel advancements within the field.

In the last section of the survey, we identify and discuss the most relevant challenges and unresolved issues, such as the difficulties in large-scale deployment, the configuration complexity and the interoperability between different IoT devices and platforms, summarizing potential research directions for the near future.

The main challenge of implementing unidirectional communication in IoT environments with high data transfer rates is the lack of a feedback mechanism for acknowledging the receipt of data. This can lead to significant packet loss, increased retransmission latency, and substantial difficulties in synchronizing devices.

Overall, this study provides a structured insight into the current status and the evolution of unidirectional communications and highlights the role of these technologies in developing secure and efficient IoT systems. Further development and optimization of the unidirectional communication solutions can provide new key directions for supporting robust IoT systems able to meet complex security requirements.

## Figures and Tables

**Table 1:** Advantages/disadvantages of hardware-based data diode solutions.

Ref.	Solution Features	Advantages	Disadvantages
[24]	Hardware–software data diode for unidirectional transmission from OT to IT. Tested on Zybo Z7 board, uses RS-485 channel.	The data diode is completely transparent between the OT and IT networks because all process data are quickly replicated into the IT network behind the data diode. PiBridge’s RS-485 channel is used to establish communication between IT and OT networks, leveraging a well-known communication standard.	The data diode has been developed and tested on the Zybo Z7 board. This may minimize its compatibility with various hardware setups, thereby limiting its usefulness in a variety of industrial applications. The paper does not specify the solution’s scalability. It is unknown whether the proposed data diode can be scaled to industrial systems or networks.
[25]	Miniature data diodes for IoT devices provide security at the network’s edge. This ensures that data flows unidirectionally over the IoT network layer.	Small data diodes incorporated into IoT devices ensure that security protocols are directly entrenched in individual devices, resulting in a more comprehensive defense against potential threats.	The integration of small data diodes into IoT devices may present technological obstacles, particularly in terms of guaranteeing seamless interoperability and operation across multiple types of devices and platforms.
[26]	This solution combines data diodes, robust encryption, and a genuine random number generator. Uses a serial port with the receive-data pin disabled, allowing for the insertion of a diode or digital buffer.	Data diodes and encryption work together to safeguard data monitoring.	This method entails purposely disconnecting the receive-data pin and establishing a direct connection, which may not be suitable for implementation in many instances and may limit the solution’s applicability in some contexts.
[27]	Data diodes separate the PV system from the internet. They also separate the home network from the photovoltaic system.	Data diode in a daisy chain ring topology network means that the attacker has to perform more investigation to reach the ring from the end of the daisy chain if one of the daisy chain rings is compromised.	The cost is the main disadvantage of the solution. The data diode is suitable for PV networks (fields) rather than residential consumers due to its higher costs. Remote access to the system is limited.
[6]	Unidirectional communication solution using a data diode and AI to protect driver’s medical data privacy. Unidirectional communication between the server and healthcare providers.	The approach reduces the risk of revealing personal information while focusing on delivering the required information for driver monitoring.	The success rate of the AI system in detecting non-compliant data may result in false positives or false negatives. This may result in unnecessary notifications or missed identification of proper concerns. The solution was tested only for denial-of-service and man-in-the-middle attacks, which may make the method vulnerable to other attacks.

**Table 2:** Advantages/disadvantages of software-based data diode solutions.

Ref.	Solution Features	Advantages	Disadvantages
[28]	Virtual data diode using distributed software-defined networking (SDN) controllers eliminates a single point of failure. It integrates directly with OSGi services and supports external interfaces such as REST, command-line, WebSockets, and a monitoring component for dynamic rule enforcement.	Distributed SDN controllers eliminate the risk of a single point of failure, enhancing system reliability and resilience. The monitoring component embedded into the virtual data diode allows for the dynamic insertion of new rules, ensuring that diode behavior is enforced for each new host added.	The virtual data diode is vulnerable to weaknesses that may not apply to its physical counterparts, posing security problems.
[29]	Software data diode using barcodes as a communication channel. Data are received unidirectionally and asynchronously from the broadcaster.	As a software solution, it is easy to develop, deploy, and maintain the method. Asynchronous communication provides an additional layer of security, making it more difficult for potential attackers to identify data exchanges.	The data transfer rate is determined by the receiving camera’s frames per second (fps). This constraint may affect the overall pace of data transfer, potentially making it slower than alternative communication methods.

**Table 3:** Advantages/disadvantages of unidirectional gateway solutions.

Ref.	Solution Features	Advantages	Disadvantages
[30]	Fiber-optic network communication for CPS security Unidirectional gateways replace firewalls to protect OT networks.	The usage of fiber-optic network communication is intended to improve the security of cyber–physical systems by offering more robust protection against potential threats. Gateways improve industrial network security by protecting control systems against external threats.	The focus on designing a secure communication architecture includes a severely confined feedback loop, which may limit the system’s responsiveness.
[31]	UNIWAY is a unidirectional security gateway with a proprietary file transmission mechanism. Two proxy systems are used to simulate FTP file transfers. Fiber-optic connectivity for “send only” and “receive only” interfaces.	Using two proxy systems, the sending proxy and the received proxy, enables successful file server/client replication. This emulation can improve the efficiency and reliability of the file transfer process.	While using the FTP protocol assures compatibility, it can also be a constraint, particularly if the protocol becomes outdated or if there are security issues associated with it. Depending on the design and execution, using proxy systems may result in single-point failures. If one of the proxy systems fails, it may impair the overall file transfer operation.
[32]	Gateway to provide unidirectional communication between OT and IT systems. FIFO-based communication method integrated with the PikeOS hypervisor. Simulates IT response and ensures secure gateway communication.	The security of the process is reinforced through the authentication of an OPC UA client, which adds an extra layer of verification to ensure the legitimacy of the communication.	Implementing security protections, authentication processes, and feedback simulations may require additional resources from the whole system, thereby compromising performance and scalability.
[33]	Unidirectional gateways for ICS protection, restricting data transfer back to the internal network. VRF as an option for smaller systems or as an alternative for unidirectional gateways.	VRF technology in routers allows for the simultaneous functioning of multiple routing tables within a single device. It isolates devices from various tables to prohibit communication even though they share the same hardware.	Unidirectional gateways may be economically impractical for smaller utility systems. The expense of acquiring and maintaining this technology could be a considerable challenge, limiting its use in some situations.

**Table 4:** Advantages/disadvantages of unidirectional protocols.

Ref.	Solution Features	Advantages	Disadvantages
[34]	A comparison of IoT data protocols (MQTT, AMQP, CoAP, XMPP) and standard techniques (Websockets, DDS). Focus on smart home automation.	MQTT and CoAP have reduced transmission times, allowing for faster communication in smart home automation. These protocols take less time to create packets, implying faster data transmission.	Each protocol serves a distinct purpose, implying that the use case or application may determine the protocol used.
[30]	Fiber-optic communication for CPS security. Uses the LLC1 and LLC2 communication protocols.	Due to the lack of acknowledgment in data diode transfers, the method of sending each message numerous times with proper identifiers, hash values, and, optionally, encryption assures reliability. LLC1 and LLC2 allow for flexibility in communication protocols depending on the system’s requirements.	The need to transmit each message many times for dependability, with the presence of identities, hash values, and possible encryption, might result in more significant network traffic and resource use.

**Table 5:** Advantages/disadvantages of network pump solutions.

Ref.	Solution Features	Advantages	Disadvantages
[12]	A battery solution is incorporated to prevent message loss during power outages.	Installing a battery into the network pump addresses the difficulty of a complete response queue buffer, increasing dependability by protecting against message loss during peak loads.	The pump’s most significant problem occurs when the response queue buffer fills up. This signals a potential bottleneck in handling message requests, which may affect the system’s responsiveness.
[36]	Two pump prototypes: E-Pump and D-Pump. E-Pump uses process ACKs, D-Pump uses TCP ACKs. E-Pump runs on the XTS-300 platform; D-Pump runs on a 486-class single-board computer.	Operating at the process layer enables the E-Pump to adjust process layer acknowledgments, perhaps resulting in more efficient and specialized processing. The D-Pump acts at the transport layer and offers a different layer perspective than the E-Pump.	It is mentioned that E-Pump implies potential issues with implementation or maintenance. Modulating Transport Layer acknowledgments (TCP ACKs) may present additional difficulties or potential concerns compared to handling genuine process acknowledgments of D-Pump.
[37]	Theoretic method to prevent covert time channels in the pump system. Buffers and injects noise into acknowledgment timings. The residual information flow rate is calculated using finite and infinite random noise.	The network pump improves security by including random noise in low-user-acknowledgment periods, potentially lowering the probability of covert transfer. Setting a maximum capacity for the communication channel serves as a control mechanism, assisting in the management and regulation of information flow.	The pump system and associated methods, such as buffers and noise injection, do not remove covert time transmission, indicating a potential gap in obtaining an enhanced security method.
[38]	The pump is described as a feedback-driven communication mechanism. Reroutes packets and acknowledgments via an intermediary node. Evaluates the upper limit of data capacity for hidden channels.	The system successfully regulates data rates by rerouting packets and acknowledgments through an intermediary node (the pump), prohibiting high Users from communicating with low Users at non-zero rates. The method considers scenarios in which the pump’s buffer is fully utilized, offering a thorough review of the system’s performance under a variety of settings.	While the pump system tries to improve security, the use of noise and data rerouting raises worries about potential weaknesses or exploitation by attackers.

**Table 6:** Analysis of areas of application areas of unidirectional solutions.

Unidirectional Solution	Ref.	Area of Application							
		Medical Systems	Industrial Automation Systems	Home Automation	Critical Infrastructure	Data and File Transfer Security	Process Control Systems	Secure Communication	Network Security
**Hardware data diode**	[6,24,25,26,27]	**✓**	**✓**						
**Software data diode**	[28,29]		**✓**						
**Unidirectional gateway**	[30,31,32,33]		**✓**		**✓**	**✓**	**✓**		
**Protocols**	[30,34]		**✓**	**✓**					
**Network pump**	[12,36,37,38]				**✓**			**✓**	**✓**

**✓**: Area of application mentioned.

## Data Availability

Data are contained within the article.

## Abbreviations

The following abbreviations are used in this manuscript:

ARP	Address Resolution Protocol
BACnet	Building Automation and Control Network
CIFS	Common Internet File System
eDNA	Enterprise Distributed Network Architecture
FTP	File Transfer Protocol
FTPS	File Transfer Protocol Secure
HTTP	Hypertext Transfer Protocol
HTTPS	Hypertext Transfer Protocol Secure
ICCP	Inter-Control left Communications Protocol
LON-IP	Local Operating Network over Internet Protocol
Modbus RTU	Modbus remote terminal unit
Modbus TCP	Modbus Transmission Control Protocol
NFS	Network file system
NTP	Network Time Protocol
OPCDA	OLE for Process Control Data Access
OSIsoft PI	OSIsoft PI System (PI Historian)
REST API	Representational State Transfer Application Programming Interface
RFTS	Owl Remote File Transfer Service
RSV	Remote screen viewing
SCP	Secure Copy Protocol
SFTP	SSH File Transfer Protocol
SMB	Server Message Block
SMTP	Simple Mail Transfer Protocol
SNMP Traps	Simple Network Management Protocol Traps
Syslog	System Logging Protocol
TCP	Transmission Control Protocol
UDP	User Datagram Protocol
WMQ	IBM WebSphere MQ (formerly known as MQSeries)
